# The role of lateral modulation in orientation-specific adaptation effect

**DOI:** 10.1167/jov.22.2.13

**Published:** 2022-02-22

**Authors:** Yih-Shiuan Lin, Chien-Chung Chen, Mark W. Greenlee

**Affiliations:** 1 Institute of Experimental Psychology, University of Regensburg, Regensburg, Germany; 2 Department of Psychology, National Taiwan University, Taipei, Taiwan; 3 Neurobiology and Cognitive Science Center, National Taiwan University, Taipei, Taiwan

**Keywords:** lateral modulation, surround inhibition, tilt-aftereffect, spatial vision, divisive inhibition

## Abstract

Center-surround modulation in visual processing reflects a normalization process of contrast gain control in the responsive neurons. Prior adaptation to a clockwise (CW) tilted grating, for example, leads to the percept of counterclockwise tilt in a vertical grating, referred to as the tilt-aftereffect (TAE). We previously reported that the magnitude of the TAE is modulated by adding a same-orientation annular surround to an adapter, suggesting inhibitory lateral modulation. To further examine the property of this lateral modulation effect on the perception of a central target, we here used center-surround sinusoidal patterns as adapters and varied the adapter surround and center orientations independently. The target had the same spatial extent as the adapter center with no physical overlap with the adapter surround. Participants were asked to judge the target orientation as tilted either CW or counterclockwise from vertical after adaptation. Results showed that, when the surround orientation was held constant, the TAE magnitude was determined by the adapter center, peaking between 10° and 20° of tilt. More important, the adapter surround orientation modulated the adaptation effect such that the TAE magnitude first decreased and then increased as the surround orientation became increasingly more different from that of the center, suggesting that the surround modulation effect was indeed orientation specific. Our data can be accounted for by a divisive inhibition model, in which (1) the adaptation effect is represented by increasing the normalizing constant and (2) the surround modulation is captured by two multiplicative sensitivity parameters determined by the adapter surround orientation.

## Introduction

The concept of lateral modulation describes how the visual percept of a central stimulus can be affected by the presence of a surround pattern. Such lateral modulation of the stimulus surround on sensitivity to the stimulus center has long been recognized (De Weerd & Pessoa, 2003; Gilbert & Wiesel, 1990). Examples of the lateral modulation effect includes visual crowding, perceptual grouping, figure–ground segregation, tilt illusion, and perceptual filling-in (Clifford, 2014; Komatsu, 2006; Schwartz et al., 2007, 2009; Solomon et al., 2004; Spillmann & Werner, 1996). At the cortical level, it is shown that even if a stimulus presented to a region outside the classical receptive field does not elicit neural activity, it can modulate the neuronal response to a central stimulus, suggesting that such lateral modulation effects extend beyond the typical classical receptive field (Barlow, 1953; Bringuier et al., 1999; Cavanaugh et al., 2002a, 2002b; Chen et al., 2001; Hubel & Wiesel, 1959; Maffei & Fiorentini, 1976). This long-range lateral modulation effect suggests that neurons responding to the center and surround regions of the visual field do not work in isolation but rather interact and integrate the activity of nearby neurons.

The lateral modulation effect has been the focus of numerous studies that implement various paradigms to investigate the observed phenomena. For example, in psychophysical experiments, the perceived contrast of a central stimulus can be decreased or enhanced by a surrounding pattern, and such a lateral modulation is tuned to the surround features such as luminance contrast, spatial frequency, size, and orientation (Cannon & Fullenkamp, 1991; Chubb et al., 1989; Meese et al., 2007, 2009; Petrov et al., 2005; Petrov & McKee, 2006; Polat & Sagi, 1993; Snowden & Hammett, 1998; Solomon et al., 1993, 1999; Yu et al., 2001, 2002). For example, Yu, Klein and Levi (2001) reported that the perceived contrast of the stimulus center was suppressed when the center and surround patterns were of the same orientation, whereas it was enhanced when the center and surround orientations were orthogonal. Such lateral modulation can differ in foveal and peripheral vision. The lateral modulation effect was found to be both facilitative and suppressive in the fovea, but was only suppressive in the periphery (Xing & Heeger, 2000). In a dual-masking paradigm, in which a Gabor target is presented with a superimposed pedestal grating of various contrast level while surrounded by flankers, Chen and Tyler (2001, 2002b) showed that colinear flankers produced a facilitative effect on the target threshold at low pedestal contrasts while they induced a suppressive effect at high contrasts. In comparison, orthogonal flankers could only produce suppressive effects on the central target threshold (also see Chen & Tyler, 2002a, 2008; Meese, Summers, Homes, & Wallis, 2007; Meese et al., 2009; Solomon & Morgan, 2000).

In addition to psychophysical experiments, single cell recording studies also provide insights into the lateral modulation effect and its properties. Many studies showed that lateral modulation from the surround depends on the spatial configuration and luminance contrast of the center and surround stimuli (Blakemore & Tobin, 1972; Kapadia et al., 1999; Levitt & Lund, 2002; Nelson & Frost, 1978, 1985; Sengpiel et al., 1998; Smith et al., 2006). Among them, Polat and colleagues (1998) manipulated the contrast of the target that was presented within the cell's receptive field of the cat as well as the contrast and orientation of two lateral flankers located outside of the receptive field. They discovered that high-contrast collinear flankers facilitated the response of the neurons to the low-contrast target and suppressed the response to high-contrast targets, whereas orthogonal flankers showed mostly a suppressive effect on the target response. In a later study, Chen and colleagues (2001) reported four types of contrast-dependent lateral modulation effects and proposed a sensitivity modulation model to explained the single-neuron recording data. Some researchers used gratings in different sizes and contrasts to estimate the summation receptive fields of neurons in macaque V1 and discovered that the summation receptive field measured at a low contrast was larger than that measured at high contrast (Li & Li, 1994; Sceniak et al., 1999; Sengpiel et al., 1997; Shushruth et al., 2009). Thus, the effect of the surround on the neuronal activity is contrast dependent, that is, it is suppressive at high contrast and facilitative at low contrast.

Neuroimaging studies have also revealed possible neural correlates of the lateral modulation effect. In one functional magnetic resonance imaging study, Williams and colleagues (2003) reported that adding a surround grating to the central pattern reduced the blood-oxygen level-dependent signal in early visual areas and that a parallel surround grating produced a stronger signal decrease, suggesting an orientation-specific lateral suppression effect. Similarly, another study (Chen et al., 2005) showed in a functional magnetic resonance imaging experiment, where a flickering pinwheel pattern was presented, that the unstimulated interwedge regions was associated with a decreased blood-oxygen level-dependent signal, indicating a lateral inhibition effect from the surrounding areas. In another functional magnetic resonance imaging study involving a lateral masking paradigm (a central grating is surrounded by collinear and noncollinear flankers), Chen (2014) further partitioned the lateral suppression effect into a more general inhibition effect insensitive to spatial features of the surround as well as a more specific effect tuned to the surround spatial configuration.

Several computational models have been proposed to account for the lateral modulation effect. Chen and Tyler (2001, 2002b) proposed a divisive inhibition (or contrast gain control) model using multiplicative sensitivity parameters to explain the flanker effect observed in their dual masking experiments. Xing and Heeger (2001) put forth a variant of the contrast gain control model to explain the center-surround modulation under different surround configurations by including weights that represent surround facilitation and suppression. Schwabe and colleagues (2006) proposed a recurrent network model that considers the top-down feedback connections in the visual cortex to account for the near- and far-surround modulation. Their model successfully predicts the contrast-dependent lateral modulation subsequently observed in macaques (Schwabe et al., 2010). In a later review, Angelucci et al. (2017) proposed a theoretical model that involved feedforward, feedback, and horizontal connections to explain the surround modulation effects.

Previous studies of center-surround modulation focused more on how the lateral interaction influences the target contrast discrimination (Chen & Tyler, 2002a; Meese et al., 2007; Xing & Heeger, 2000) or perceived contrast (Cannon & Fullenkamp, 1991; Yu et al., 2001, 2002) and less on how the target orientation percept can be affected by an adapter modulated by surround features. Many neurons in the visual cortex are tuned to stimulus orientation and neurons tuning to neighboring orientations can inhibit each other (Blakemore et al., 1970; Blakemore & Tobin, 1972; Carpenter & Blakemore, 1973; Hubel & Wiesel, 1962, 1968); thus, it is also important to explore the effect of lateral modulation in the orientation percept.

The tilt illusion and the tilt-aftereffect (TAE) are two visual phenomena commonly studied to understand the orientation domain of human vision (Clifford, 2014; Gibson & Radner, 1937). The tilt illusion describes the situation when a surround oriented-grating alters the perceived orientation of a center grating to the opposite direction of the surround orientation, whereas the TAE demonstrates that the perceived orientation of a target grating can be tilted away from a preceding oriented adapter. In both cases, the effect is the strongest when the surround pattern or the adapter has orientation close to (10°–20° away from) the center stimulus or the target, indicating that neurons tuned to similar orientations inhibit each other, which results in the visual illusion and aftereffect of a tilt adaptation. Magnussen and Kurtenbach (1980) reported that adding a second adapting pattern ranging from 6° to 60° clockwise (CW) to a 15° CW adapter decreased the TAE on the subsequently presented vertical target. The authors concluded that a lateral inhibition process was involved because the inhibitory effect from neurons inhibiting the vertical (and near vertical) orientation channels caused by the first adapter was inhibited by the second adapter (see also Kurtenbach & Magnussen, 1981). Similarly, Greenlee and Magnussen (1987, 1988) implemented a method of sequential adaptation in which two adapting patterns were alternating in time during the adaptation phase and estimated the contrast threshold of a following target grating. Their results showed that, when the two adapting gratings were of the same spatial frequency or orientation, the target threshold increased, suggesting a suppressive effect. In contrast, the contrast threshold decreased as the second adapting grating deviated from the first in spatial frequency and orientation and even went below the level when only one adapter was presented, indicating an inhibitory effect between the two adapting patterns. Adaptation effects involving patterns with multiple oriented components provided further insights in how gratings interact with each other. For example, the plaid pattern composed of two oblique sinusoidal gratings can be perceived as a blurred checkerboard of horizontal and vertical edges. Such compound checkerboards became distorted after adaptation to one oblique grating, suggesting that the adaptation process interfered with the combination and interaction between responses to the two oblique gratings resulting from combining responses of two orientation filters (Georgeson, 1992; Georgeson & Meese, 1996). Later, Meese and Georgeson (1996a, 1996b) used plaids and gratings varied in orientation and contrast as adapters and recorded whether the participants perceived the test plaid as a compound pattern or individual components. They found that the adapter plaid with a 45° rotation from the test plaid, but of the same spatial frequency decreased the percentage of the compound response, whereas the aligned adapter plaid, that is, having the same orientation but with a spatial frequency that was three times higher, increased the percentage of the compound response. These results show that the filter combination process after adaptation depends on the difference in spatial configuration between the adapter and the test stimulus. In addition, adaptation to a vertical and horizontal grating made the test plaid look stretched horizontally and vertically, indicating the presence of a TAE in the compound percept. These studies demonstrated the power of the adaptation paradigm in revealing how two patterns overlapping spatially interact with each other. Here, our study focused instead on the interactions between patterns not overlapping in space; that is, how the surround pattern affects the neural response to the center pattern. Thus, we adapted the TAE paradigm to further investigate how the center and surround regions of the adapter could interact with each other and inferred the lateral modulation effect during the adaptation phase.

In a previous study (Lin et al., 2020), we selectively adapted the center, the surround, and both the center and the surround regions using a center-surround sinusoidal grating as an adapter in the periphery and estimated the magnitude of the TAE on the target. We used three types of adapters: a center adapter that had the same spatial extent as the target, a disk adapter that covered both the center and surround regions, and an annulus adapter that was located in the surround region without physical overlap with the target. We found that the TAE was most pronounced for the center adapter, intermediate for the disk adapter, and weakest for the annulus adapter. The decrease in the TAE magnitude of the disk condition compared with the center condition indicated an inhibitory lateral modulation effect from the adapter surround. The limitation of the previous study is that in the disk condition, the adapter surround always had the same orientation as the adapter center. Therefore, we could not capture how the lateral modulation effect on the adapter center would change if the adapter surround was of a different orientation, which now is the focus of the current study.

Studying the effect of the adapter surround orientation on the adapter center allows us to observe how the TAE changed quantitatively with varying surround orientations, leading us to investigate the cross-orientation interaction of the lateral modulation effect in the orientation domain. Therefore, to further investigate the property of such a lateral modulation effect here, we manipulated the surround and center orientations independently. By doing so, we can observe how much the lateral modulation effect from the adapter surround was induced on the adapter center by measuring the changes in TAE on the subsequently presented target. In an adaptation paradigm, a center-surround adapter was presented followed by a target Gabor, about which participants were to make an orientation judgement. We measured how the TAE induced on the target depended on the adapter center and surround orientations. Because many earlier studies have shown that such lateral modulation depends on the surround features, we expect to observe an orientation-specific modulation on the TAE as the surround orientation is varied. A contrast gain control (or divisive inhibition) model has been shown to be able to explain the lateral modulation effect reported in psychophysics experiments (Chen & Tyler, 2001, 2002b; Clifford, 2014; Goddard et al., 2008; Meese et al., 2007; Schwartz et al., 2009; Xing & Heeger, 2001) Therefore, we fitted a modified divisive inhibition model inspired by previous studies (Chen & Tyler, 2001, 2002b; Foley & Chen, 1997; Lin et al., 2020) to our data and examined how the model parameters capturing the lateral modulation effect varied with changes in the surround orientation. If the lateral modulation effect is independent of the surround orientation, then the TAE magnitude should remain constant regardless of the variation in the surround orientation. In contrast, if the lateral modulation effect is feature specific, then the TAE magnitude should be different across conditions of different surround orientations.

## Methods

### Participants

Four observers, aged between 20 and 30 years, including one of the authors (Y.S.L., referred to in the following as P0) and three participants naïve with respect to the purpose of the experiment (P1–P3) participated in the study. All observers have normal or corrected-to-normal vision. Informed consent was acquired before participation for all participants. The study procedure and protocols were approved by the University of Regensburg ethics committee (application number: 19-1591-101) and the experiment was performed according to the Declaration of Helsinki on human experimentation. Participants (except for P0) received monetary compensation or class credits as a reward for their participation. All observers first performed a short practice session to become acquainted with the stimuli and the task before continuing the formal experiment.

### Apparatus

Participants viewed stimuli on a Dell S2417DG 24-inch LED monitor with 2560 × 1440 pixel resolution and 120 Hz refresh rate in a viewing distance of 60 cm. The monitor was calibrated and gamma-corrected with a spot photometer (MINOLTA CS-100). The mean luminance was 73.8 cd/m^2^. The experiment was conducted in a dimly lit room.

### Stimuli

**Figure 1.**
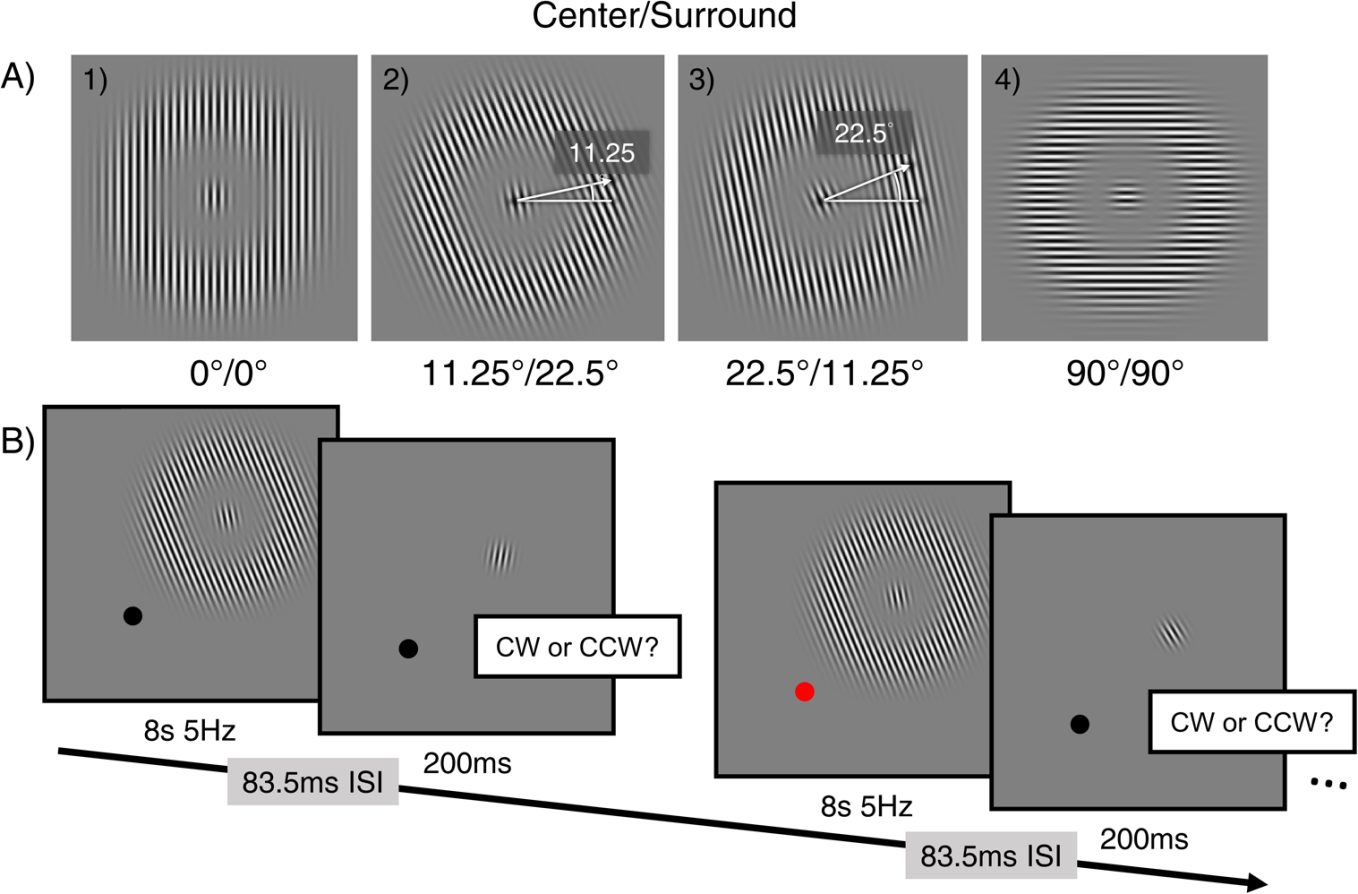
(A) Example stimuli used in the current study. The adapter consists of two gratings: a central patch (having the same spatial extent as the target) and a surround annulus. (A)Four of the 25 adapters with varying center and surround orientation. The corresponding orientation of the two gratings are indicated below each panel in center/surround format. The white arrows (presented here only for the sake of illustration) in panels A2 and A3 signify the orientation of the central patch with 11.25° and 22.5° of CCW tilt, respectively. (B) An illustration of the experimental procedure. The stimuli were all presented in the upper-right visual field. The black dot represents the fixation point (not scaled to actual size). During the adaptation phase, the fixation point briefly changed its color (from black to red and back to black) at random timepoints. Participants were instructed to press a button to report the color change. See text for more details.

The adapter was composed of two parts: a center Gabor pattern (the center patch) and a surround grating (the annulus). The stimulus orientation here was defined by a sinusoidal luminance contrast variation, with 0° corresponding to a vertical grating and CW gratings was assigned negative values whereas counterclockwise (CCW) gratings, positive values. The orientation of the center and surround gratings varied independently of each other along one of five orientations (0°/vertical, 11.25°, 22.5°, 45° and 90°/horizontal) in separate runs, resulting in 25 possible orientation combinations. Figure 1A shows some examples of the adapters. The target was a Gabor with the same spatial extend of the center patch, defined by:

(1) $$G\left(x,y\right)=B+BCcos\;\left(2\pi fx^{'}\right)e^{\left(\frac{-x^{{}^{'}2}-{\left(y^{'}-u_{y}\right)}^{2}}{2\sigma^{2}}\right)}\;$$

$$x^{'}=xcos\;\theta \;+ysin\;\theta ,$$

$$y^{'}=-xsin\;\theta \;+ycos\;\theta ,$$

where *B* represents the mean luminance, *C* the pattern contrast, *f* the spatial frequency, *μ_y_* the vertical displacement of the pattern, and *σ* the scale parameter. *θ* in the second and third equations represents the pattern orientation. The center part of the adapter and the target had a 0.3° scale parameter (*σ*). The annular surround part of the adapter was generated by multiplying a sinusoidal grating by an annular Gabor envelope, defined by:

(2) $$G_{a}=B+BCcos\;\left(2\pi fx^{'}\right)\cdot \;e^{\left(\frac{-{\left(r-r_{E}\right)}^{2}}{2{\sigma_{r}}^{2}}\right)},$$

where *r* = (*x*′^2^ + *y*′^2^)^0.5^ is the radial coordinates of *x*′ and *y*′ after transformation from Cartesian coordinates to polar coordinates. *r_E_* is the eccentricity which determines the size of the annulus, whereas σ_*r*_ is the radial scale parameter that determines the width of the annulus. The annular surround part used in the current study was created with 3.5° eccentricity (*r_E_*) from the adapter center with a 0.9° scale parameter (σ_*r*_). There was no overlap between the adapter center (where the center patch and the target locate) and the adapter surround (where the annulus is positioned) regions.

The adapter and the target were presented on the upper right quadrant of the visual display, centered at 10° eccentricity (7.07° in the *x* and *y* directions) from the central fixation point. The visual stimuli were all generated using Matlab (MathWorks, Inc., Natick, MA) with PsychToolbox (http://psychtoolbox.org/).

### Procedure

A single interval binary choice task was used to estimate how much the percept of the target orientation appeared tilted following adaptation. In every run, the adapter had 1 of the 25 orientation combinations. Each orientation combination was repeated at least three times. The sequence of the orientation combination was randomly determined. Each run contained 72 trials, including two practice trials at the very beginning. On each trial, the adapter flickered at 5Hz in counterphase for 8 seconds, followed by an 83.3 ms interstimulus interval then the target, which lasted for 200 ms (see Figure 1B for an illustration of the procedure). Observers were instructed to judge the target orientation (CCW or CW) by pressing the corresponding keyboard buttons. The next trial began automatically after the response. Two types of targets were presented in random sequence in the experiment: a CCW-tilted or a CW-tilted one. To determine the placement of the orientation of either target in the next trial, we used the Ψ threshold-and-slope-seeking staircase (Kontsevich & Tyler, 1999) method and created one staircase for each type of target to estimate the orientation level necessary for the observer to judge the target as oriented in the same direction as its physical orientation at 86% rate. In each staircase, if the observer judged the target as oriented the same as the appearance in a previous trial, the target orientation in the next trial became closer to the vertical orientation; Otherwise, the target orientation deviated more away from the vertical orientation in the next trial.

**Figure 2.**
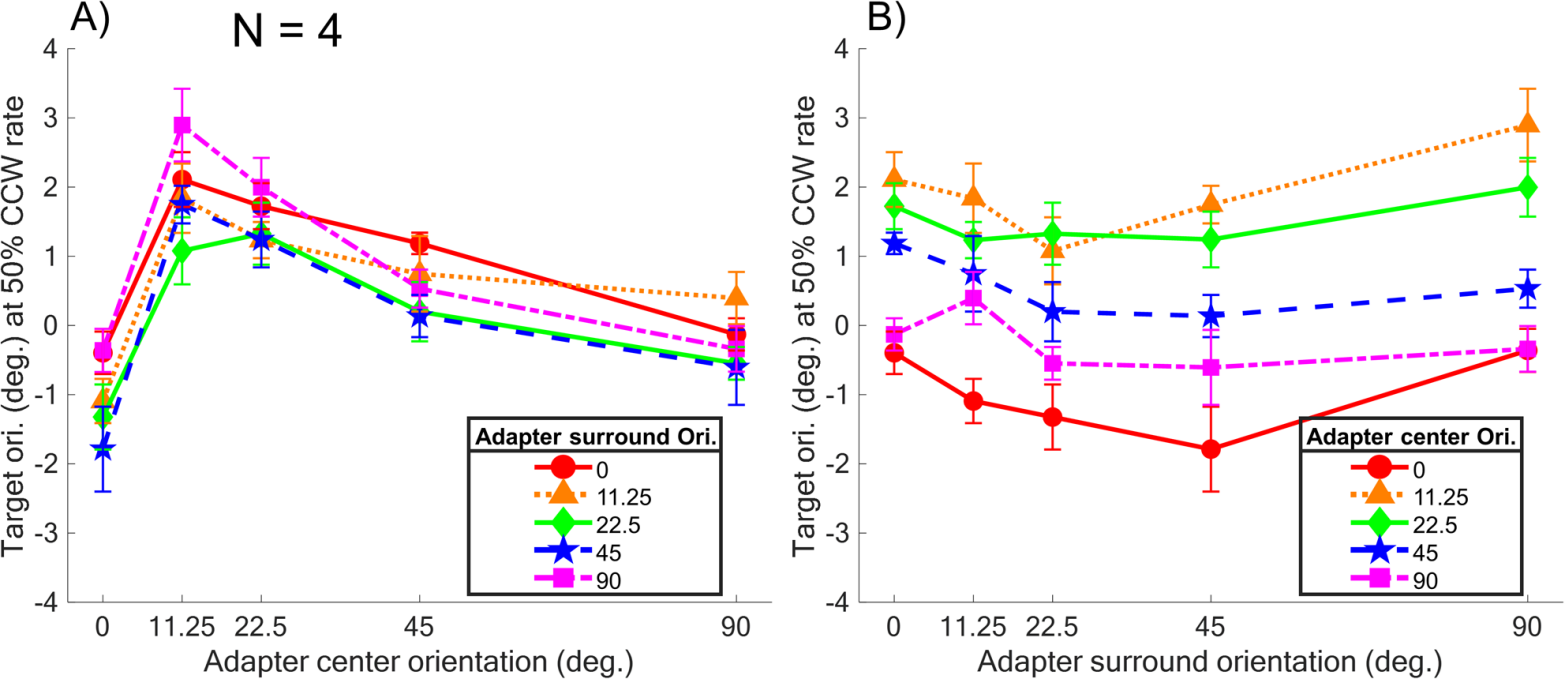
The averaged TAE (corresponding to a 50% CCW response rate of the four observers) is shown. In (A), the TAE is plotted against different adapter center orientations with different color symbols and curves representing different surround orientations; in (B), the TAE is instead plotted against different adapter surround orientations, whereby the different color symbols and curves represent different center orientations. The error bars are ±1 standard error of measurement.

To make sure observers maintained steady fixation during the task, we added a central fixation task, in which they were instructed to press the space bar whenever the color of the fixation dot turned from black to red (see Figure 2B). All participants were trained to reach a high accuracy (>95%) in the fixation task during the practice session before we started the real experiment.

## Results

We combined the CW and CCW trials of each orientation combination condition together and fit one psychometric function (PF) to the combined data (see Lin, Chen, & Greenlee, 2020 for a comparison between the TAE estimated by the Ψ method and the PF fitting method). We first pooled all trials of all runs of each condition for every observer, and assigned CW trials with negative orientation values, whereas the CCW trials with positive ones. We then calculated the proportion of the observer responding CCW at all orientation levels. We used the Palamedes toolbox (Prins & Kingdom, 2018) to fit a cumulative normal Gaussian PF to every adapter condition. Alpha (50% CCW response rate, essentially the point of subjective verticality) and beta (slope at 50% CCW response rate) were set as free parameters, whereas gamma (guessing rate) and lambda (finger error rate) were fixed with the value 0.01. We took the alpha values, or the point of subjective verticality, of the fitted PF of each condition as the magnitude of the TAE and determined how such values varied with different adapter orientations. In the following, the magnitude of the TAE is expressed in degrees of orientation tilted away from the physical vertical for the target to be perceived as vertical.

The data (including the Ψ estimates and the raw trial data) of individual participants are presented in Supplementary File S1 and File S2 in .xlsx (Excel) format.

### Orientation-specific lateral modulation

Figure 2 shows the averaged data of the four participants (the individual data are included in the Supplementary File S3). In panel A, the TAE is plotted against the adapter center orientation and different color curves and symbols represent different surround orientations; in contrast, in panel B, the TAE is plotted against the adapter surround orientation. The data suggest that the TAE peaked between 10° to 20° center orientation, regardless of the surround orientation and that the surround orientation modulated the overall adaptation effect. The surround modulation resulted in a dipper shape trend shown in Figure 2B, where the TAE first decreased then increased as the surround orientation increased from 0° to 90°. Such a trend is present in the data of all participants (see Figure S1 in Supplementary File S3). The lateral modulation observed in the data can be captured by sensitivity modulating parameters in a modified divisive inhibition model shown in the later section. A two-way repeated measures analysis of variance showed that the interaction between center and surround orientation, *F*(16, 72) = 1.36, *p* = 0.19, was not significant, whereas the adapter center and surround main effects, *F*(4, 72) = 77.52, *p* < 0.01, $\hat{f}$ = 1.75; *F*(4, 72) = 8.09, *p* < 0.01, $\hat{f}$ = 0.53, were significant. The significant main effects suggest that the both the center and surround orientations affect the target TAE magnitude.

To further investigate the lateral modulation effect observed in the psychophysical data where the TAE highly depended on the surround orientation, in the following section, we fitted the data with a divisive inhibition model inspired by previous studies (Chen & Tyler, 2002b; Foley & Chen, 1997).

### Model

We implemented a divisive inhibition model in which the response to a visual stimulus is computed by dividing the excitatory component with an inhibitory component plus a normalizing constant. Researchers have long used divisive inhibition models to explain lateral modulation effects such as lateral masking (Chen & Tyler, 2001, 2002b; Meese et al., 2007; Xing & Heeger, 2001) and tilt illusion (Clifford, 2014; Goddard et al., 2008; Schwartz et al., 2009). Prior studies have also used divisive inhibition models to fit the results of adaptation experiments (Foley & Chen, 1997; Meese & Holmes, 2002; Wilson & Humanski, 1993). In the current study, we would like to integrate these two aspects, the lateral modulation and the adaptation effect, in our model, modified from the one used in our previous study (Lin et al., 2020), in which the lateral modulation effect was captured by two multiplicative sensitivity (or gain) modulating parameters (Chen & Tyler, 2002b), whereas the adaptation effect was represented by changes in the normalizing constant (Foley & Chen, 1997).

#### Model architecture

**Figure 3.**
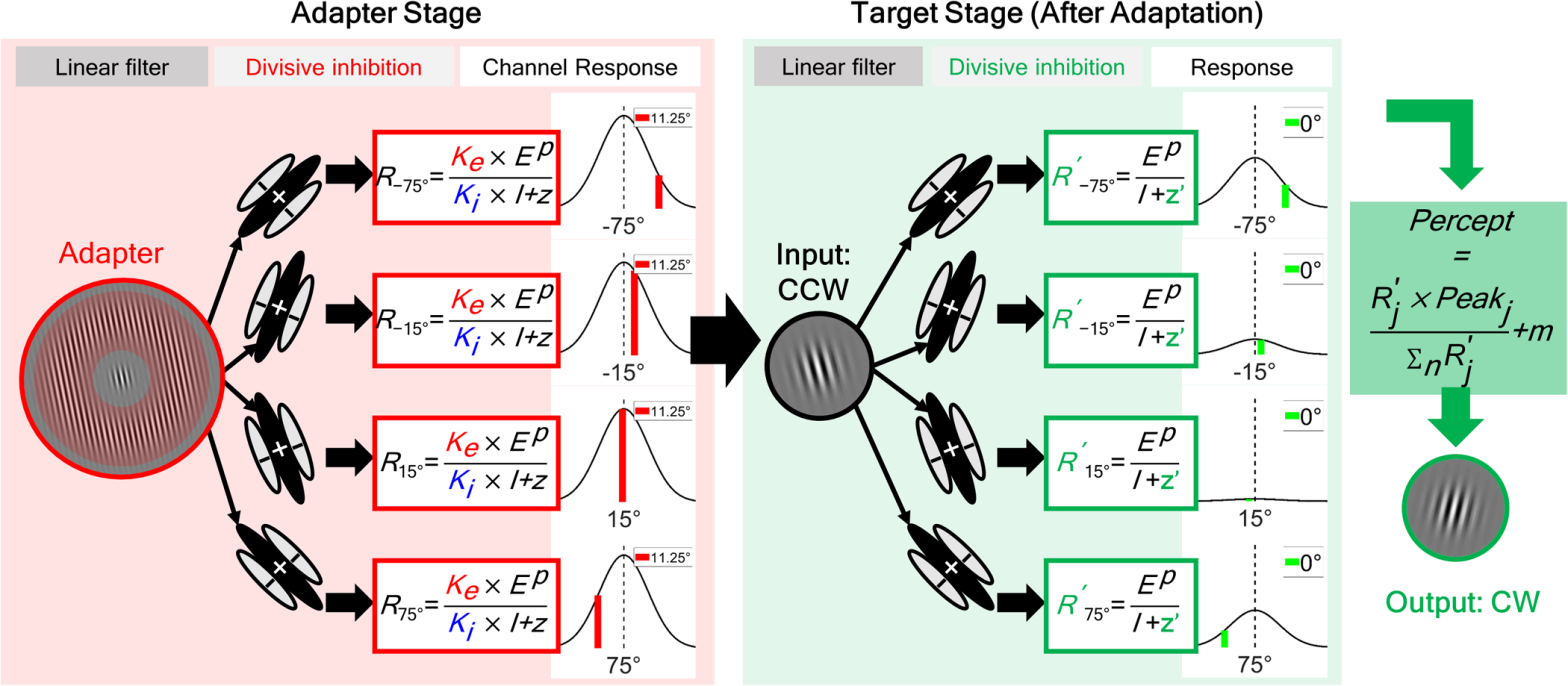
A schematic diagram of the divisive inhibition model. In this population coding model, each channel response to the input image is calculated by a contrast gain control process. The adapter response determines the adaptation effect induced on the target. The lateral modulation effect is captured by two sensitivity modulation parameters, *K_e_* and *K_i_*. See the text for further details.

In the model (illustrated in Figure 3), we implemented population coding (Deneve et al., 1999; Paradiso, 1988; Pouget et al., 2000) and assumed multiple evenly distributed orientation channels with preferred orientation ranging from −90° (CW) to 90° (CCW) at 30° interval and 30° full width at half maximum. The response of the *j^th^* channel to the *i^th^* image first goes through a receptive field-like linear operator (the oriented-receptive field/linear filter in Figure 3), then a nonlinear operator (the divisive inhibition process in Figure 3).

The excitatory component of channel *j* is calculated as the product of the sensitivity profile of the linear operator and the image *i* (Chen et al., 2000; Foley & Chen, 1999; Phillips & Wilson, 1984). The sensitivity profile is assumed to be a Gabor function that matches the Gabor pattern used in the experiment (see the section on Stimuli in the Methods section). Integrating the product of the sensitivity profile and the stimulus over space, we end up with the following three components shown in Equation 3: *C_i_*, the luminance contrast of *i^th^* image (which is independent of the image spatial structure, thus is taken out as a separate term), the orientation dependent component (i.e., the orientation-tuning function) that can be represented by a Gaussian function (Paradiso, 1988; Pouget et al., 1998; Westrick et al., 2016; Wilson & Humanski, 1993), and finally the orientation independent part of the product that is defined as a constant in the current case. This last constant component is termed the sensitivity parameter, *Se*. Combined, the excitation component thus is defined as,

(3) $$E_{ij}^{'}=Se\cdot C_{i}\cdot e^{-\;\frac{{\left(\theta_{i}-\theta_{j}\right)}^{2}}{\sigma^{2}}},$$

where θ_*i*_ is the image orientation and θ_*j*_ the channel preferred orientation. σ^2^ is the channel variance determining the channel bandwidth. If a surround region is added to the center, as is the case for the center-surround grating adapter, the excitation can be modified as the following,

(4) $$\begin{array}{ccc} E_{ij}^{'} & = & \;E_{icj}^{'}+\;E_{isj}^{'}=Ke\times (Se_{c}\cdot C_{ic}\cdot e^{-\;\frac{{\left(\theta_{ic}-\theta_{j}\right)}^{2}}{\sigma^{2}}}\\ & & +\;Se_{s}\cdot C_{is}\cdot e^{-\;\frac{{\left(\theta_{is}-\theta_{j}\right)}^{2}}{\sigma^{2}}}), \end{array}$$

in which the center and surround parts of the image belong to separate components, $E_{icj}^{'}$ and $E_{isj}^{'}$ with *C_ic_* and θ_*ic*_ representing the features of the image center, whereas *C_is_* and θ_*is*_ represent those of the image surround. *Se_c_* and *Se_s_* are the excitatory sensitivity parameters for the two regions. Parameter *Ke* is included to capture the lateral modulation effect from the surround to the center. The excitation term is then halfwave rectified, as in many previous studies (Chen & Tyler, 2001, 2002b; Foley, 1994; Foley & Chen, 1997, 1999), shown in Equation 5, where max(*a*,  *b*) indicates the operation of choosing the larger value among *a*,  *b*.

(5) $$E_{ij}=max\left(E_{ij}^{'},\;0\right),$$

Note that, in the current experiment, because no terms in Equation 3 or 4 are negative, the halfwave rectification transformation can be skipped without changing the excitatory component value. However, we retain such a step to align with previous studies and to keep the model flexible for future cases in which negative terms could be involved.

Next, before adaptation, the excitation component is raised by a power, *p*, and divided by the inhibitory component, *I_ij_*, as well as the normalizing constant, *z*, as

(6) $$R_{ij}=\;\frac{{E_{ij}}^{p}}{I_{ij}+z}\;,$$

where *I_ij_* is

(7) $$I_{ij}=Si\cdot {\left(E_{ij}\right)}^{q},$$

for images without a surround pattern with *Si* the inhibitory sensitivity parameter, and

(8) $$I_{ij}=Ki\times \left(Si_{c}\cdot {\left(E_{icj}\right)}^{q}+Si_{s}\cdot {\left(E_{isj}\right)}^{q}\right),$$

when the surround region is added, where contributions from the center and surround are represented again by separate components (with individual sensitivity parameters *Si_c_* and *Si_s_*); the sum of the two is then multiplied by the lateral modulation parameter, *Ki*.

*Ke* and *Ki* are determined by two Gaussian functions of the surround orientation (θ_*is*_).

(9) $$Ke=e^{-\;\frac{{\left(\theta_{is}-\theta_{excitatory}\right)}^{2}}{\sigma_{excitatory}^{2}}}\;\text{and}\;Ki=e^{-\;\frac{{\left(\theta_{is}-\theta_{inhibitory}\right)}^{2}}{\sigma_{inhibitory}^{2}}},$$

Each Gaussian function has a mean (the θ_*excitatory*_ or θ_*inhibitory*_) and standard deviation parameter (the σ_*excitatory*_ or σ_*inhibitory*_) to determine the center orientation and bandwidth of the function. These four center and bandwidths parameters control how the excitatory and inhibitory sensitivities vary with surround orientation.

We determine the perceived orientation of the input image, *i*, with a population coding operation where the preferred orientation of each channel, θ_*j*_, is weighed by the channel response, *R_ij_*, then divided by the sum of all channel responses, as

(10) $$P_{i}=\;\frac{\sum_{j=1}^{N}R_{ij}\cdot \;\theta_{j}}{\sum_{j=1}^{N}R_{ij}}+m.$$

An internal bias parameter *m* is included to account for the perceptual or response bias of each observer even without experimental manipulations such as adaptation.

The effect of adaptation has been shown by the shift of the dynamic range of V1 neuron contrast response function (Albrecht et al., 1984; Anderson et al., 1997; Gardner et al., 2005; Sclar et al., 1989) and, in a Naka-Rushton style model (Naka & Rushton, 1966), can be modeled by introducing a change in the semisaturation parameter (Greenlee & Heitger, 1988). In psychophysics results, such an adaptation effect can be captured by the change of parameter, *z*, the normalizing constant (Foley & Chen, 1997; Lin et al., 2020). Thus, after adaptation is induced, the channel response, denoted by a prime, ', superscript, to the target becomes the following:

(11) $$R_{targetj}^{'}=\;\frac{{E_{targetj}}^{p}}{I_{targetj}+z_{j}^{'}}=\;\frac{{E_{targetj}}^{p}}{I_{targetj}+\;z\cdot a_{j}},$$

where $z_{j}^{'}$ is the normalizing constant after adaptation and *a_j_* serves as the adaptation factor, defined as,

(12) $$a_{j}=\left(1+R_{adapterj}\right).$$

The *R_targetj_* is calculated by Equations 3, 5, 6, and 7, and *R_adapterj_* is determined by Equations 4, 5, 6, and 8, in which lateral modulation components are involved.

#### Model performance

**Figure 4.**
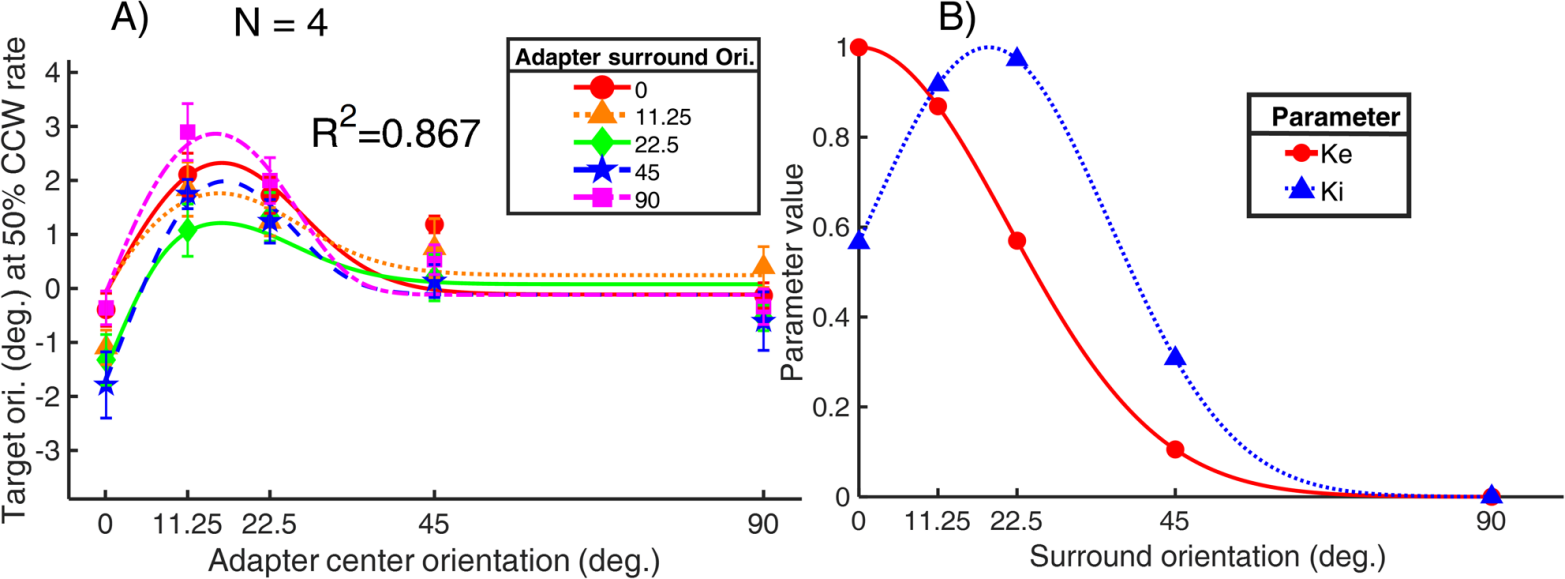
Model fitting results of the averaged data. In (A), the target orientation, corresponding to a 50% CCW response rate, is plotted against the adapter center orientation. Smoothed curves of different colors represent the model prediction for the different adapter surround orientations (inset), whereby the differently colored symbols show the averaged TAE data in different adapter surround orientation conditions (inset). The error bars are ±1 standard error of measurement. (B) How the parameters *Ke* and *Ki* vary with the surround orientation. Both parameters were determined by a Gaussian function.

We fitted the aforementioned model to the averaged data (Figure 2) with Powell's algorithm (Press et al., 1988), which seeks the parameter values that minimize the sum of squared error, or the sum of the squared deviations between the TAE data and the model prediction. The smooth curves in Figure 4 represent the best model prediction. The symbols in Figure 4 are the averaged TAE data as shown in Figure 3.

In the model, *Se* (excitatory sensitivity to the target) and *Se_c_* (excitatory sensitivity to the adapter center) are set to be the same since the target has the same spatial extent as the adapter center. Likewise, *Si* (inhibitory sensitivity to the target) and *Si_c_* (inhibitory sensitivity to the adapter center) are set to be equal. Parameters other than *z*,  *p*,  *q*, *m*, *Si_s_*, and the two parameters determining *Ki* were fixed because the goodness of fit did not change empirically whether they were free or not, resulting in a total of seven free parameters. The model can explain up to 86.7% of the variance in the averaged data, with a root mean square error of 0.43. The goodness of fit ranged from 70.5% to 89.0% in the four participants. The best fitting parameters and goodness of fit of the group-averaged data and the individual data are presented in Table S1. In Supplementary File S3, the plots for the fitting results of the individual participant can be found in Figure S2.

As demonstrated in Figure 4B, the parameter *Ke*, controlling how the excitatory sensitivity is modulated when a surround is added to the adapter, decreased as the surround orientation increased from 0° to 90°. In contrast, *Ki*, the inhibitory sensitivity modulating parameter, peaked at approximately 20° and then decreased as the surround orientation continued to increase. These results suggest that the surround interaction in the numerator terms of the normalizing process was stronger when the surround orientation was closer to 0°, whereas the surround modulation in the denominator was stronger with about 20° deviation from vertical.

## Discussion

Magnussen and Kurtenbach (1980) demonstrated that adding a second oriented adapting pattern decreased the TAE on the subsequent target, suggesting a inhibitory lateral interaction between the two patterns (see also Greenlee & Magnussen, 1987, 1988). In the current study, we investigated the cross-orientation interaction in center-surround oriented gratings by manipulating the orientation of the adapter center and surround and observed its effects on the TAE. In an adaptation paradigm, we implemented a center-surround sinusoidal grating adapter that contains a center and surround regions whose orientations varied independently. We then measured how the TAE induced by the adapter on a following target changed with adapter center and surround orientation. We found that the overall TAE was determined by the orientation of the central adaptor, peaking at approximately 10° to 20°, and that the magnitude of the TAE is modulated by the surround orientation. In general, the TAE first decreased then increased as the surround orientation deviated away from the vertical orientation (the dipper shape in Figure 2B). Our results demonstrates that the surround interaction is orientation specific, as has been reported in the literature (Cavanaugh et al., 2002b; Chen & Tyler, 2002b; Shushruth et al., 2012; Solomon et al., 1993).

Such orientation-specific lateral suppression that we observed has been compared to the overlay cross-orientation suppression in which a mask usually of orthogonal orientation was superimposed on the target. Petrov and colleagues (2005) measured the target detection threshold under surround masking and overlay masking configurations. They reported that the surround suppression effect was more narrowly tuned to the mask features such as orientation and spatial frequency and was more evident in the periphery than in the fovea. Similarly, Meese et al. (2009) examined the target contrast threshold under three masking configurations: cross-oriented overlay mask, orthogonal surround mask, and parallel surround mask at different eccentricity and mask contrast. Again, the surround suppression, especially from the parallel surround mask, was found to be stronger in the periphery, whereas the superimposed cross-oriented masking remained at similar strength across foveal and peripheral locations. Such findings suggest that these two forms of suppression involve different neural processes (see also Petrov & McKee, 2006; Smith et al., 2006).

### Divisive inhibition in orientation-specific lateral modulation

To explain such orientation-specific lateral modulation effects, we modified the divisive inhibition model to account for our data. In the previous model implemented in the our earlier study (Lin et al., 2020), the sensitivity-modulating effect was mediated by the excitatory sensitivity parameter, *Se*, which was most pronounced for the center, intermediately pronounced for the disk (both center and surround) and was least pronounced for the annulus (surround-only) condition in the final fitting results.

In Chen and Tyler (2002b), *Ke* and *Ki*, the multiplicative sensitivity modulating parameters of the excitatory and inhibitory components, were defined as free parameters. They reported that *Ke* and *Ki* both decreased as the flanker orientation increased while the ratio between the two stayed approximately constant with flanker orientation. They fitted each of the two parameters with a linear combination of two Gaussian functions, one narrowly and one broadly tuned. In our model, *Ke* and *Ki* were each determined by a Gaussian function, representing how the excitatory and inhibitory terms change with the surround orientation, respectively (see Figs. 4B and S3). Similar to the fitting results of Chen and Tyler (2002b), in our case, the best fitting parameter set showed that *Ke*, peaking at the 0° surround, decreased as the surround orientation increased, whereas *Ki* first increased and peaked around 20°, then decreased as the surround orientation increased. These studies demonstrated that the modified divisive inhibition model can capture not only the lateral modulation effect of the flankers on the contrast threshold of a central stimulus in the dual-masking paradigm (Chen & Tyler, 2002b), but also the lateral modulation effect of the adapter surround orientation on the percept of the adapter center in the adaptation paradigm (Lin et al., 2020 and the current study).

### Comparison with the results of Lin et al. (2020)

In our previous study (Lin et al., 2020), we measured the TAE on the target after three types of adapter: a center adapter with the same spatial extent as the target, a disk adapter that occupied both the center and surround regions, and an annulus adapter that covered only the surround region. We found that the TAE was more pronounced in the center adapter condition than in the disk adapter condition, suggesting that adding a surround to the adapter introduced an inhibitory effect to the adapter center. In this section, we compare the lateral modulation effects estimated from the previous and the current studies.

The reasons to make such a comparison are two-fold: 1) to see if we could replicate the previous lateral inhibition result and 2) to examine how introducing a gap between the center and surround could affect the lateral modulation effect. Because the surround region of the disk adapter in the 2020 study always had the same orientation as the adapter center, a fair comparison condition from the current study would be when the adapter center and surround have the same orientation. Three participants (P0, P1, and P3) took part in both the 2020 study and the current one; thus, we could compare their data from the two studies.

**Figure 5.**
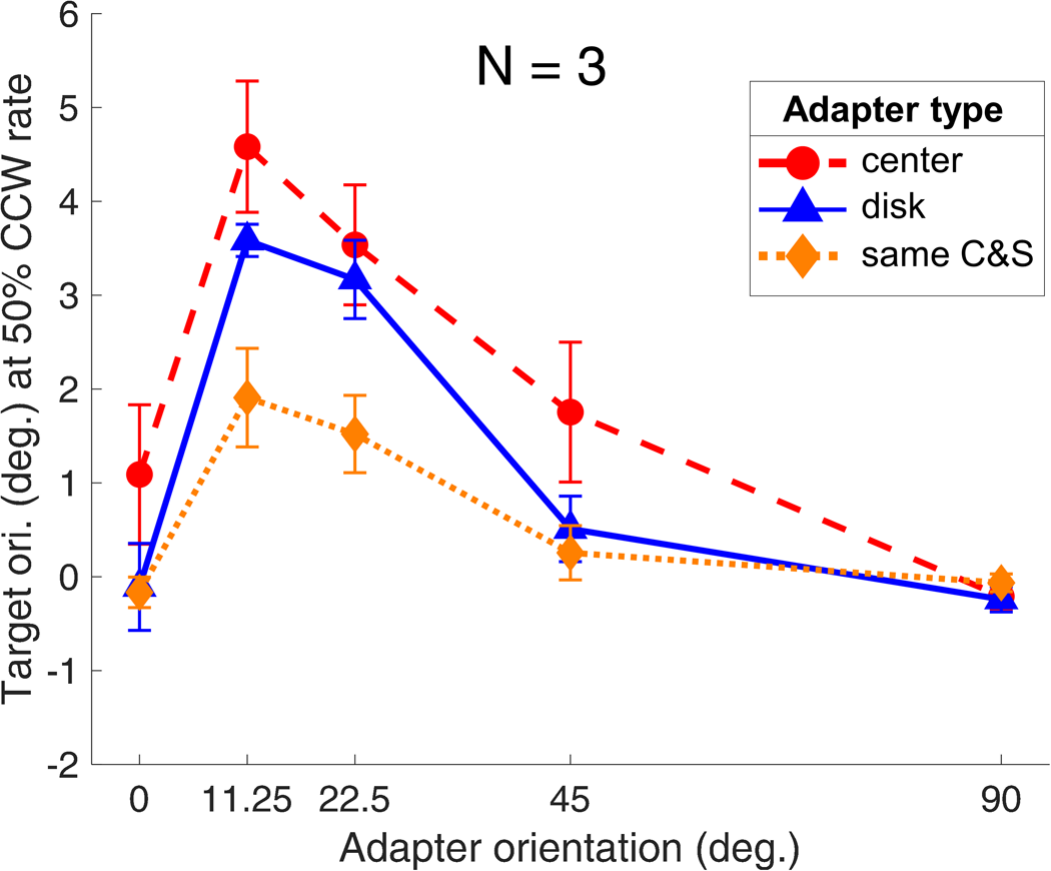
Comparison between the same center and surround (C&S) conditions of the current study and the center and disk conditions from the previous study (Lin et al., 2020). The averaged TAE data of three participants (P0, P1, and P3) are plotted against the adapter orientation. The red-dashed curve with red solid circles represents the center, the blue curve with solid triangles presents the results from the disk adapter (an enlarged grating covering both the center and the surround area), and the orange-dotted curve with solid diamonds depicts the results of the center-surround adapter when both regions had the same orientation. The error bars are ±1 standard error of measurement.

We took the data of the center and the disk adaptation conditions from the 2020 study and compared these data with the conditions where the adapter center and surround were of the same orientation (same center and surround in Figure 5) in the current study. Because the data reported in Lin et al. (2020) were based on the psi estimates, whereas the data shown in the current study were the psychometric function (PF)-fitting results, we also fitted PFs for the center and disk conditions on the raw data of the 2020 study and took the 50% CCW reporting rate target orientation for comparison purposes. As shown in Figure 5, the TAE was the most pronounced in the center condition, followed by the disk condition, and it was least pronounced in the center-surround condition with the same orientation. Thus, we again demonstrated that including a surround pattern during the adaptation decreased the TAE on the target. The individual data of three participants of the comparison between the two studies are shown in Figure S4 in Supplementary Materials.

Comparing the conditions where the adapter center and surround had the same orientation (same center and surround condition in Figure 5) in the current study with the results from the center and the disk conditions in the previous 2020 study, we replicated the effect showing that adding a surround decreased the TAE. Interestingly, the TAE in the disk condition was more pronounced than that exhibited in the same center and surround conditions, suggesting that adding a gap between the adapter center and surround increased the lateral inhibition effect from the surround, thereby decreasing the magnitude of the resultant TAE. One possibility is that the grating filling in the gap region in the disk condition induced a near surround interaction that facilitated the adapter center and countered the surround inhibition from the more eccentric surround region (the annulus) thus resulted in a slightly stronger TAE compared with the same center-surround condition.

Introducing a gap between the center and surround regions of a stimulus could affect the amount of lateral modulation the center region received. In the tilt illusion, segmenting the center and surround gratings by adding a mean luminance ring in between, separating the two with different disparity, or changing the relative contrast decreases the perceived illusion (Durant & Clifford, 2006; Qiu et al., 2013). The perceived contrast decrease of a center grating surrounded by a grating ring also decreased as the physical distance between the ring and the center patch increased (Cannon & Fullenkamp, 1991; Yu et al., 2001). In a flanker paradigm, the target contrast detection threshold first increased (suppression) then decreased (facilitation) with the target-to-flanker distance (Polat & Sagi, 1993). Chen and Tyler (2008) manipulated the relative location of the flankers and the flankers distance to the target. The facilitative and suppressive flanker effects decreased as the flanker location deviated from the collinear axis. When the flanker-to-target distance was shortened (1.4 λ), the flankers acted like a high-contrast pedestal and it raised the target threshold regardless of the pedestal contrast. As the distance between the flankers and the target further increased, the target detection threshold further decreased. In Meese et al. (2009), the maximum contrast threshold elevation resulting by adding an annulus surround mask decreased as the gap between the annulus and the center Gabor increased. These findings indicate a complex and diverse effect of the segregation between the center and surround. In our case, future experiments manipulating the separation between the center patch and the annulus would be required before further inference can be made on the effect of the gap on the lateral modulation.

## Conclusions

Previously, Lin, Chen, and Greenlee (2020) showed that adding a surround with the same orientation to the adapter center decreased the TAE magnitude of the subsequently presented target, suggesting a lateral inhibition effect. Here, we further investigated such lateral modulation effects by varying the center and surround orientations separately.

In an adaptation paradigm, participants were asked to judge the orientation of a central Gabor target (located in the upper right periphery) after viewing a flickering sinusoidal-grating adapter with a center (occupying the same spatial extent as the target) and a surround (without physical overlap with the target) gratings in eccentric vision. Because the center and surround orientations were varied independently, we could observe how the surround feature could influence the center percept quantitatively. The results showed that the TAE induced on the target was predominantly determined by the adapter center and modulated by the adapter surround. The surround modulation effect first increased then decreased as the surround orientation deviated from the vertical orientation. Such findings demonstrate an orientation-specific interaction between the center and the surround regions in the visual field. The results aligned well with previous research on the lateral modulation effect in human vision (Chen & Tyler, 2001, 2002b; Magnussen & Kurtenbach, 1980). We fitted a divisive inhibition model to the data, using the sensitivity modulating parameters in the numerator and denominator to capture the observed lateral modulation effect.

Numerous studies have focused on the cross-orientation interactions between patterns occupying the center and surround visual fields. The results of these studies have shed light on the neural mechanisms of the long-range interactions (Chen & Tyler, 2001, 2002b; Meese et al., 2007; Solomon & Morgan, 2000; Spillmann & Werner, 1996; Xing & Heeger, 2000; Yu et al., 2001, 2002), providing insights into the fundamental neural processing of early visual cortex. Following this long line of research in lateral interactions, the current study furthers our understanding of the human visual system by showing that the magnitude of the lateral modulation is influenced by the surround orientation.

## Supplementary Material

Supplement 1

Supplement 2

Supplement 3

Supplement 4
