# Supplementary material for: The role of lateral modulation in orientation-specific adaptation effect
Source: J Vis. 2022 Feb 22;22(2):13. doi: 10.1167/jov.22.2.13 (PMC8883160; doi:10.1167/jov.22.2.13)
Supplement: Supplement 3 [file jovi-22-2-13_s003.pdf]

Supplementary materials

This file contains the TAE data (50% CCW response points) and fitting results of all participants. Figure S1 shows the TAE data of each observer in two views, as is the case in the Figure 2 of the main manuscript. Readers can find the model fitting results of each observer in Figure S2 and S3. Figure S4 represents the data of three conditions: the center, the disk and the same C&S adapter from the current study and the previous study (Lin, Chen, & Greenlee, 2020).

Orientation-specific lateral modulation

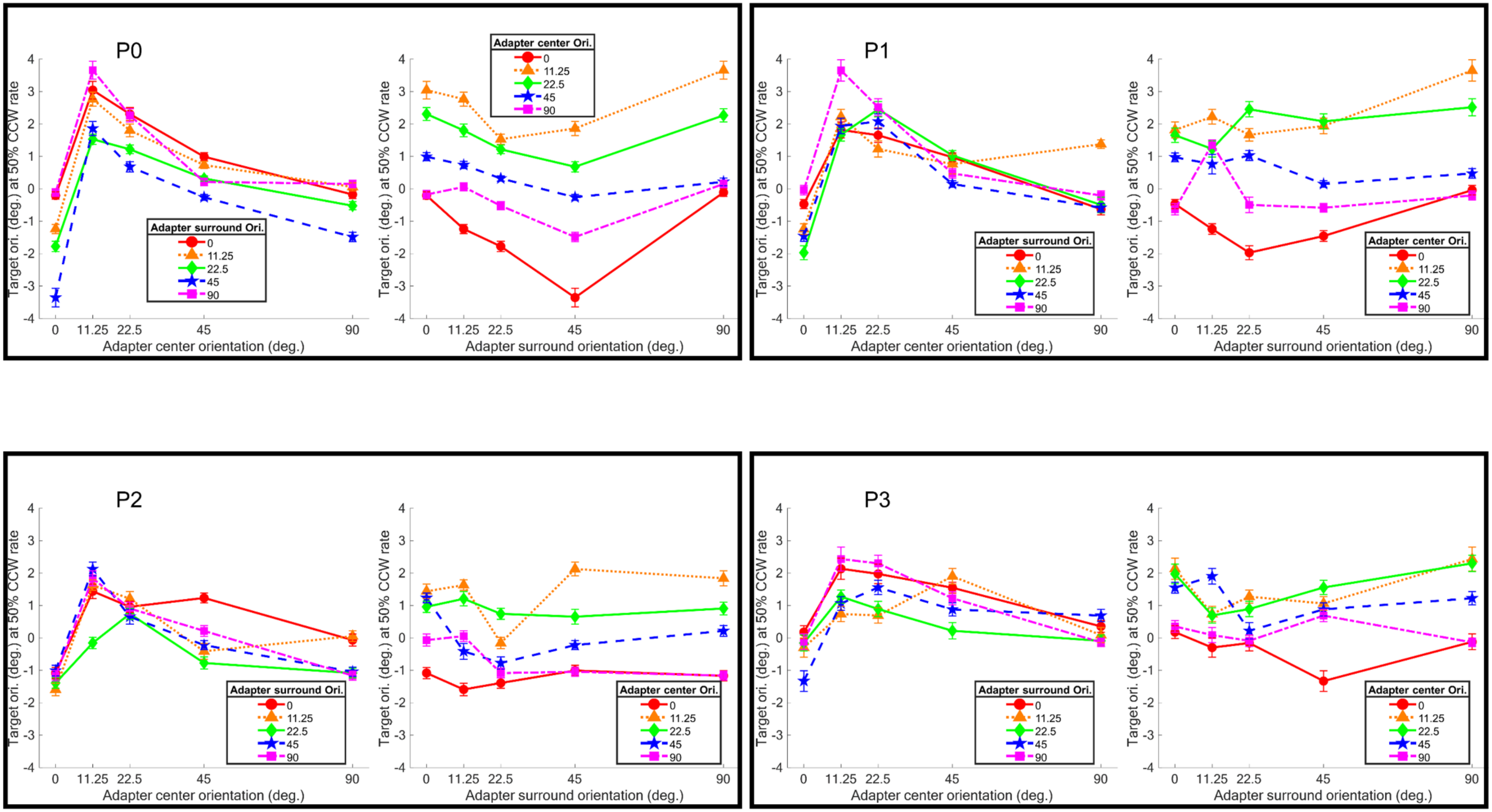

Figure S1. The data of the individual observer. Each subplot (labeled with participant's initial) presents the data set of one participant. In each subplot, the left panel shows the TAE plotted against the adapter center, whereas the right panel the TAE against the adapter surround. The error bars are ±1 standard error of measurement. See the **Results** section of the main manuscript and Figure 2 for further details.

Model fitting results of individual participants

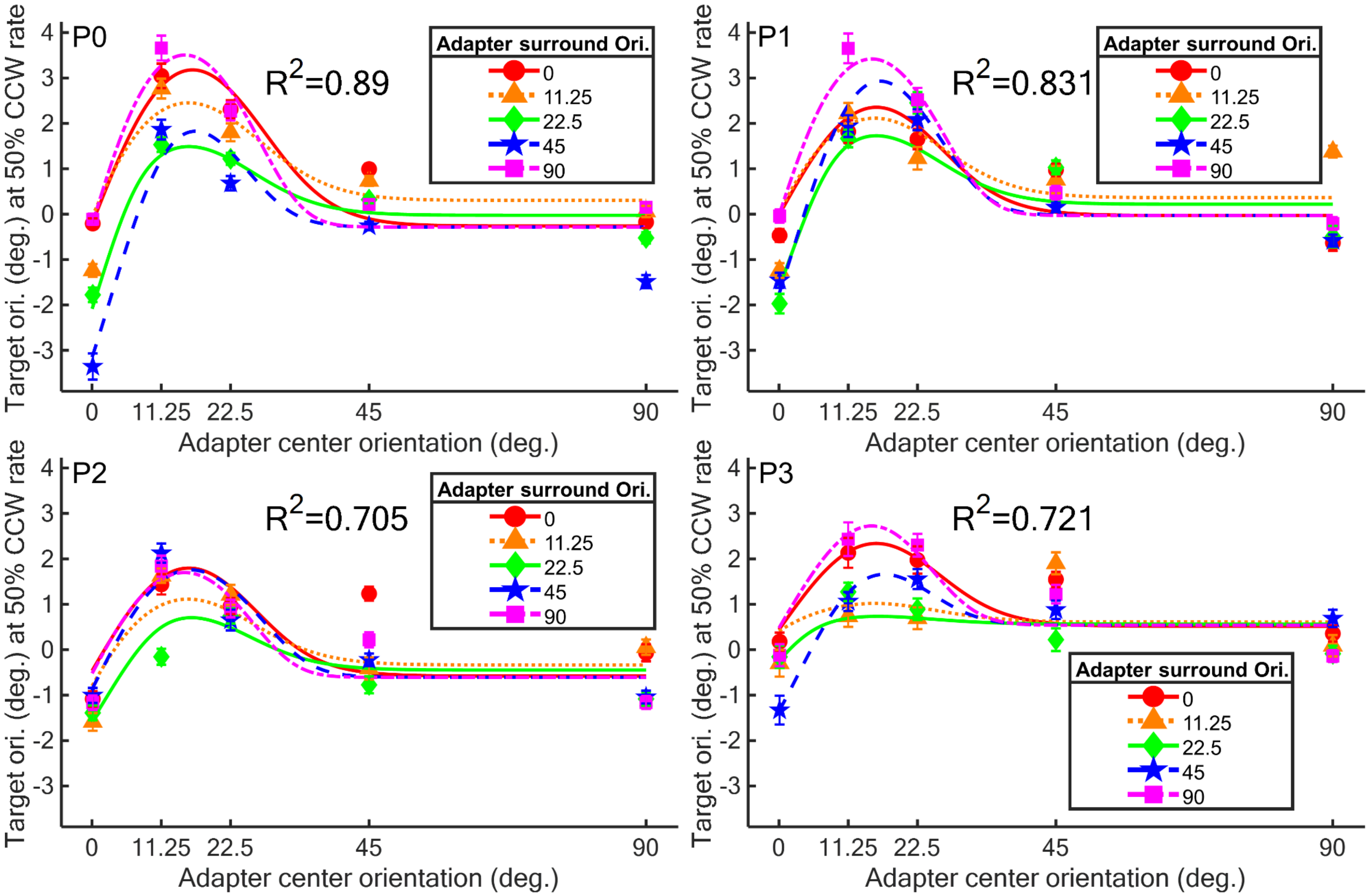

Figure S2. Model predictions of individual observer data. This figure demonstrates the fitting results of each participant. Each panel shows the model predictions (in colored-smoothed curves) and the TAE data (the colored markers). Different colors represent different adapter surround orientation.

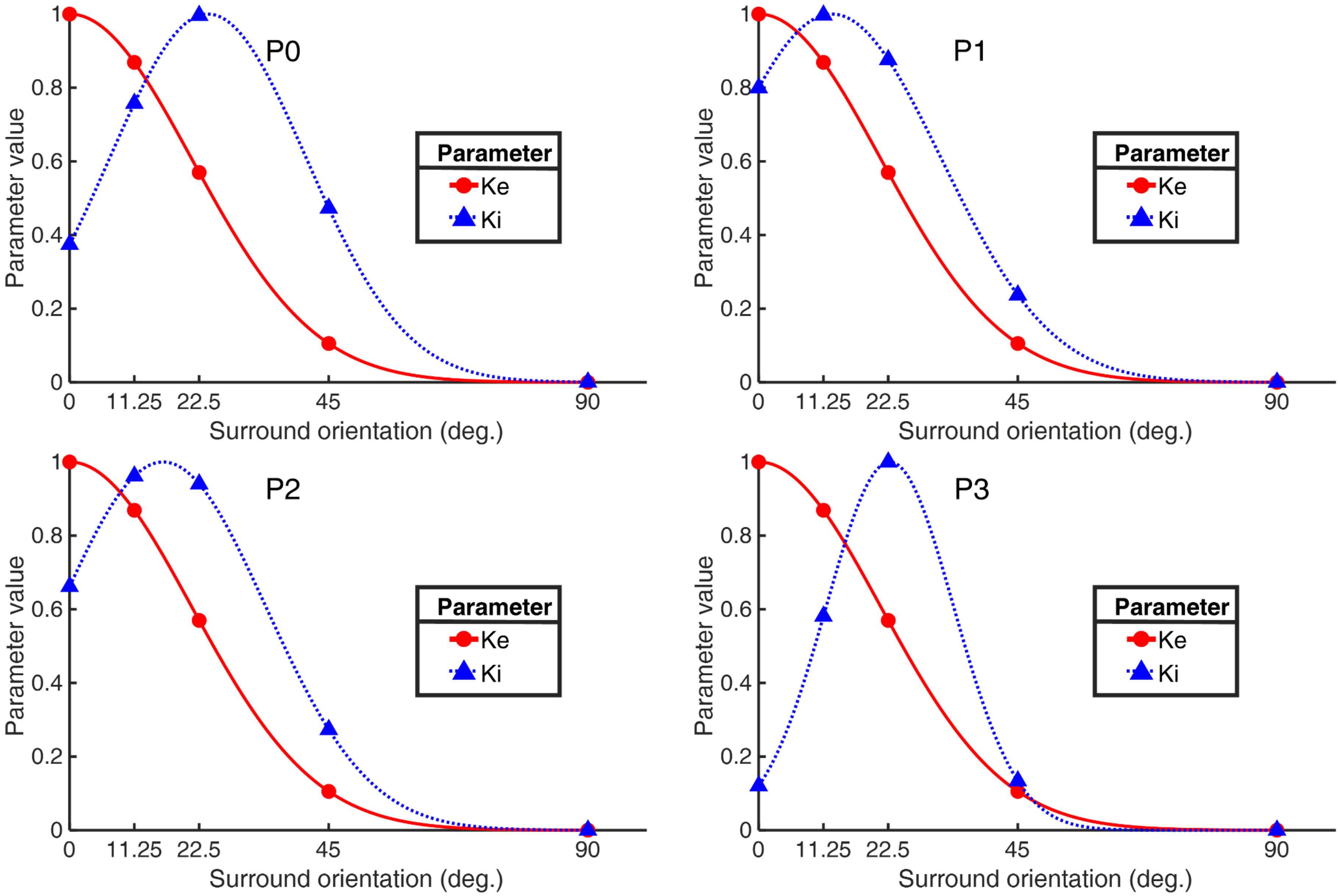

Figure S3. This figure shows fitting parameters *Ke* and *Ki* of each observer. *Ke* (red circles and solid curve) and *Ki* (blue triangles and dashed curve) are plotted against surround orientation. Colored symbols represent the surround orientations used in the experiment.

Replication of the lateral inhibition

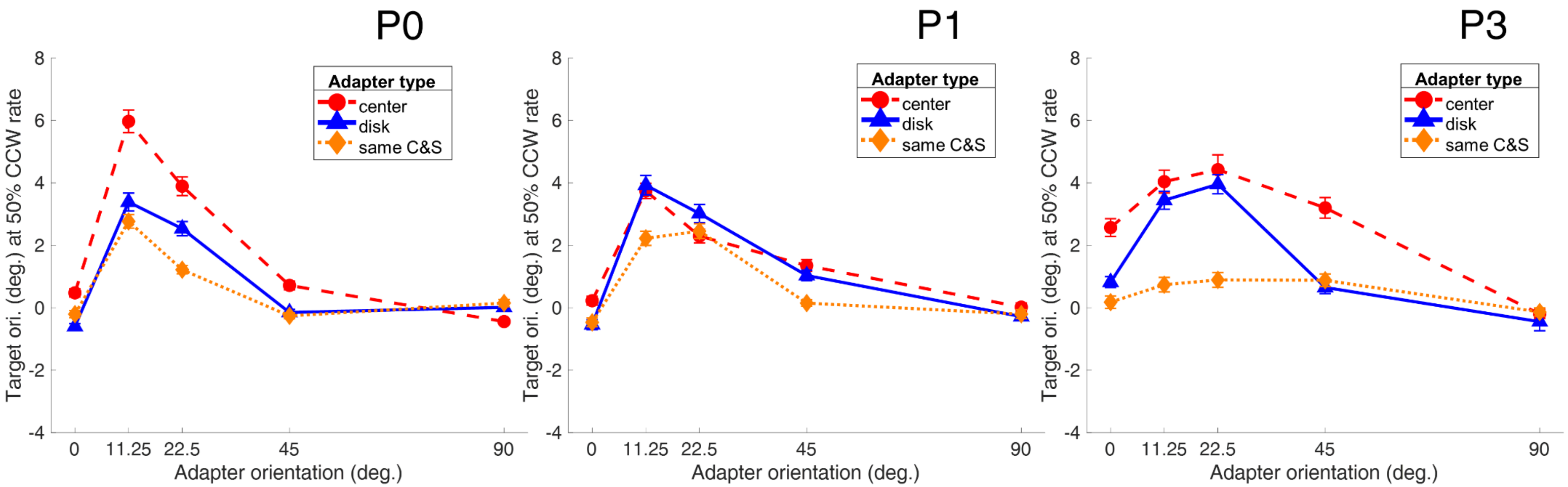

Figure S4. Comparison between data in the current study and the 2020 study of individual observers. Different colored curves and markers represent different adapter conditions. The error bars are ±1 standard error of measurement. See Figure 5 in the main manuscript and **Results** section for a more detailed description.
