# Supplementary material for: The role of lateral modulation in orientation-specific adaptation effect
Source: J Vis. 2022 Feb 22;22(2):13. doi: 10.1167/jov.22.2.13 (PMC8883160; doi:10.1167/jov.22.2.13)
Supplement: Supplement 4 [file jovi-22-2-13_s004.pdf]

Table S1

List of best fitting parameters and goodness of fit (R^2) of the current model on the averaged and individual TAE data.

Observer		MEAN	P0	P1	P2	P3
**Parameter**						
Se/Se_c		100.00	100.00	100.00	100.00	100.00
Se_s		100.00	100.00	100.00	100.00	100.00
Si/Si_c		0.20	0.20	0.20	0.20	0.20
Si_s		**17.31**	**49.55**	**17.77**	**8.62**	**44.73**
z		**0.02**	**0.05**	**0.02**	**0.07**	**0.03**
p		**2.88**	**3.02**	**3.09**	**3.21**	**2.79**
q		**1.19**	**1.18**	**1.28**	**1.63**	**1.32**
σ		18.01	18.01	18.01	18.01	18.01
Ke	$\theta_{excitatory}$	0.00	0.00	0.00	0.00	0.00
	$\sigma_{excitatory}$	30.00	30.00	30.00	30.00	30.00
Ki	$\theta_{inhibitory}$	**18.45**	**24.02**	**12.72**	**16.23**	**22.79**
	$\sigma_{inhibitory}$	**24.46**	**24.24**	**26.91**	**25.25**	**15.63**
m		**0.28**	**0.73**	**0.07**	**1.33**	**-1.09**
R^2		0.867	0.890	0.831	0.705	0.721
$RMSE$		0.43	0.53	0.57	0.60	0.49
MSE		0.38	0.16	0.20	0.18	0.23
number of free parameters (total parameters)		**7**(13)	**7**(13)	**7**(13)	**7**(13)	**7**(13)

Notes. Free parameters and parameter values are highlighted in bold font. Parameter σ was set as 18.01 to maintain the channel tuning FWHM as 30 degrees. The column **MEAN** represents the fitting parameters of the averaged data across the four participants.
